# Modeling the flux of metabolites in the juvenile hormone biosynthesis pathway using generalized additive models and ordinary differential equations

**DOI:** 10.1371/journal.pone.0171516

**Published:** 2017-02-03

**Authors:** Raúl O. Martínez-Rincón, Crisalejandra Rivera-Pérez, Luis Diambra, Fernando G. Noriega

**Affiliations:** 1 Centro de Investigaciones Biológicas del Noroeste (CIBNOR), CONACYT, La Paz, Baja California Sur, México; 2 Centro Regional de Estudios Genómicos (CREG), UNLP, La Plata, Buenos Aires, Argentina; 3 Department of Biological Sciences, Florida International University, Miami, Florida, United States of America; 4 Biomolecular Science Institute, Florida International University, Miami, Florida, United States of America; Universita degli Studi di Camerino, ITALY

## Abstract

Juvenile hormone (JH) regulates development and reproductive maturation in insects. The *corpora allata* (CA) from female adult mosquitoes synthesize fluctuating levels of JH, which have been linked to the ovarian development and are influenced by nutritional signals. The rate of JH biosynthesis is controlled by the rate of flux of isoprenoids in the pathway, which is the outcome of a complex interplay of changes in precursor pools and enzyme levels. A comprehensive study of the changes in enzymatic activities and precursor pool sizes have been previously reported for the mosquito *Aedes aegypti* JH biosynthesis pathway. In the present studies, we used two different quantitative approaches to describe and predict how changes in the individual metabolic reactions in the pathway affect JH synthesis. First, we constructed generalized additive models (GAMs) that described the association between changes in specific metabolite concentrations with changes in enzymatic activities and substrate concentrations. Changes in substrate concentrations explained 50% or more of the model deviances in 7 of the 13 metabolic steps analyzed. Addition of information on enzymatic activities almost always improved the fitness of GAMs built solely based on substrate concentrations. GAMs were validated using experimental data that were not included when the model was built. In addition, a system of ordinary differential equations (ODE) was developed to describe the instantaneous changes in metabolites as a function of the levels of enzymatic catalytic activities. The results demonstrated the ability of the models to predict changes in the flux of metabolites in the JH pathway, and can be used in the future to design and validate experimental manipulations of JH synthesis.

## 1. Introduction

Mosquito-transmitted parasitic diseases are among the major causes of mortality in the world. The wide-spread resistance of mosquitoes to insecticides underscores the need for new control approaches based on a better understanding of mosquito biology. Juvenile hormone (JH) regulates reproductive maturation in female insects [1]. JH is biosynthesized *de novo* by the *corpora allata* (CA), a pair of endocrine glands connected to the brain [2]. The biosynthetic pathway of JH III in mosquitoes involves 13 discrete enzymatic reactions which are divided into early (mevalonic acid pathway—MVAP) and late (JH-branch) steps. The early steps of JH III biosynthesis follow the mevalonate pathway to form farnesyl pyrophosphate (FPP) [3]. The late steps involve the hydrolysis of FPP to farnesol (FOL), followed by oxidation to farnesal (FAL) [4] and farnesoic acid (FA) [5]. FA is finally converted to JH III by the activity of a methyl transferase [6, 7], and a P450 epoxidase [8, 9].

Female mosquitoes show dynamic changes in JH synthesis, which have been related to their reproductive physiology [10, 11]. Studies in several insect species have shown a correlation between JH biosynthesis and the expression and activities of JH biosynthetic enzymes [12, 13, 9]. Increases or decreases in the catalytic activities of the JH biosynthetic enzymes are generally concurrent with increases or decreases in JH synthesis [10]. Furthermore, it has been demonstrated that exogenous additions of precursors stimulate JH biosynthesis in a developmental-dependent mode [14, 9]; indicating that the enzymes of the JH pathway are frequently in excess. Studies of the levels of all JH III precursors in *Aedes aegypti* CA revealed global changes in the metabolite pool sizes, and identified four developmental switches that alter JH synthesis by modulating the flux at distinctive points in the pathway [10].

A model for the regulation of juvenile hormone titers in hemolymph have been proposed for two insect species, a cricket and a moth [15]. In that model, JH titer was determined by three factors: JH rate of synthesis, rate of degradation, and the degree to which JH is protected from degradation by binding to a diversity of JH-binding proteins [15]. Although transport and degradation are important factors, numerous studies indicate that JH biosynthesis is a major regulator of JH titer. It is also widely accepted that JH is not stored in the CA and therefore the amount of JH “released” to the incubation medium or hemolymph accurately represents the amount of JH synthesized [16]. Tightly concomitant changes in JH biosynthesis and JH hemolymph titers have been described during the gonotrophic cycle of female mosquitoes [17].

A key step toward further understanding the regulation of CA activity is the establishment of numerical models that could describe and predict how changes on the individual metabolic reactions in the JH pathway affect JH synthesis. In this study, we applied two different quantitative approaches that using experimental data on enzymatic activities and/or metabolites concentrations modeled variations in the flux of metabolites in the JH pathway: 1) generalized additive models (GAMs) that are regression models used to describe non-linear relationship between response and explanatory variable(s), and 2) ordinary differential equations (ODEs) that are mathematical models that allows us to gain insight on the temporal behavior of metabolites concentrations in the JH pathway using enzymatic activities as parameters.

## 2. Materials and methods

### 2.1. Databases on enzymatic activities and intermediate metabolites data

The experimental data on enzymatic activities and CA metabolite concentrations, used for model building (S1 Table) and validation analyses (S2 Table), were obtained as previously reported [10]. Briefly, data are from female pupae and adult *Aedes aegypti* mosquitoes. Enzymatic activities were measured using “*in vitro*” assays that evaluated the capacity of CA extracts to metabolize MVAP and JH-branch intermediates, and are expressed as fmol/h/CA. Metabolite concentrations are the sizes of the precursor pools detected in CA extracts, and expressed as fmol/CA.

### 2.2. Generalized additive models (GAM)

#### 2.2.1. Definitions

In classical linear regression models, response variables (dependent variable) are continuous, and predictor variables (independent variables) can be a collection of continuous or categorical variables [18]. Linear models are used to explore linear relationship between responses and predictor variables; however, these relationships are often not linear. In this work, Generalized Additive Models (GAM) were used to describe the relationship between predictor variables and response variables. GAM are regression models involving a sum of smooth functions of covariates [19]. These functions allow to describe the shape (linear or non-linear) of relationships between responses and predictor variables. A general representation of a GAM can be written as:

$$g\left(\text{μ}\right)=\alpha +f_{1}X_{1}+f_{2}X_{2}+f_{3}X_{3}\;\ldots \;+f_{n}X_{n}$$

Where *g* is a smooth monotonic link function, μ is the expected value of the response variable, *α* is the intercept, *f* are the smooth functions, and *X* are the predictor variables.

#### 2.2.2. Model variables

Two variables were established to construct our models: 1) response variable (P), which were the reaction products within the JH biosynthesis pathway, and 2) predictor variables, which included the substrate concentrations (S) and, when available, the reaction velocities or activities of the biosynthetic enzymes (E). A general and simple representation of the GAM can be written as:

$$P=\alpha +f_{1}\left(S\right)+f_{2}\left(E\right)$$

Where *P* is the expected value of the reaction product within the JH biosynthesis pathway, *f* are the smooth functions (thin plate regression spline) [20], *S* is the observed substrate concentration, and *E* is the reaction velocity of the enzyme. All GAMs were fitted in R (R Core Team, 2015) version 3.2.0, using the mgcv package version 1.8–6 [20], assuming a Gaussian family distribution for the response variable.

#### 2.2.3. Model building procedure

There are two common approaches to obtain the “minimal adequate model”, also described as the “best-fitted model”, which includes the most relevant predictor variables: 1) to first build the maximal model (containing all the predictor variables), and subsequently remove non-significant predictor variables at each step (backward stepwise progression) [19], 2) the alternative is to first build the minimal model (null model), and afterwards add a single predictor variables at each step, measuring its contribution to the previous model (forward stepwise progression) [21]. The null model (just one parameter, the overall mean) is a conceptual model used in regression analyses to get the full variation (deviance) of the data. The criterion to include a new predictor variable in the null model is based on the reduction of deviance (i.e. explaining the variation of the response variable). In this work, the forward stepwise progression approach was used to get the best-fitted models.

Akaike information criterion (AIC) [19] and F test or chi-squared test [22] are two statistical approaches frequently used to assess the statistical significance of a decrease in residual deviance that results when a given predictor variable is added to the preceding model. In this work, AIC was used to determinate if adding a new variable increased the fitness of the model, with the best-fitted models showing the lower AIC value.

#### 2.2.4. Interpretations of GAMs

Interpretations of GAM results were performed using partial dependence plots, which describe a predictor’s contribution (i.e. the shape of the relationship) to the fitted model. Partial dependence plots represent the effect of a predictor variable on the response variable [23].

#### 2.2.5. Model validation

To validate the statistical models, we utilized a new set of experimental data that were not used to construct the original models. Predictions of concentrations of metabolites were visualized using partial dependence plots. Validating experimental data points on metabolite concentrations and enzymatic activities were integrated into the model plots.

### 2.3. Ordinary Differential Equations models (ODE)

**Fig 1:**
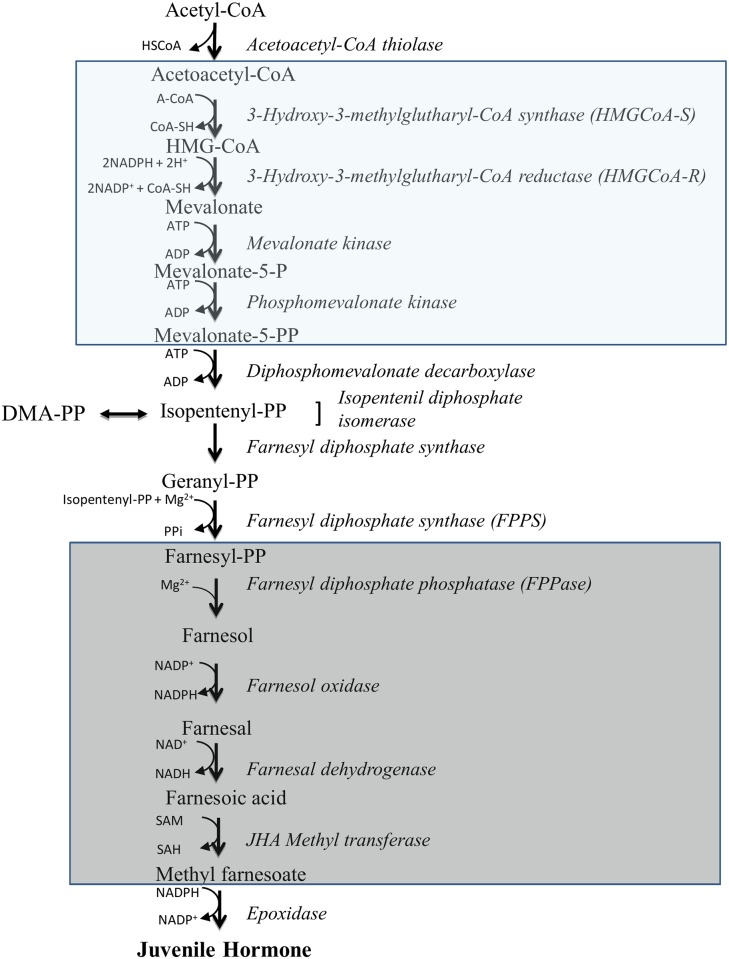
JH biosynthesis pathway: Mevalonic pathway and JH synthesis branch metabolites and enzymes. Boxes show the part of the pathways that were modeled using ODEs.

We used two different sets of ordinary differential equations to model the temporal behavior of precursors, intermediate metabolites and final products synthesized in defined segments of the MVAP and JH-branch (Fig 1). For the MVAP, data were available for the activities of 3 enzymes (HMG−CoA synthase, mevalonate kinase and phosphomevalonate kinase), which allowed us to use a set of 4 ODEs to model a segment of the MVAP that involves the conversion of aceto-acetylCoA to diphospho-mevalonate. Because the activity of HMGCoA-R that converts HMGCoA into mevalonate was not known, we had to take the liberty of considering the transformation of aceto-acetylCoA directly into mevalonate. The dependent variables in the model were AaCoA (aceto-acetylCoA), Mev (mevalonate), Mev5P (phospho-mevalonate) and Mev5PP (diphospho-mevalonate). This set of ODEs can be written as:

$$\frac{d\text{AaCoA}\left(t\right)}{dt}=\;-k_{HMGS}\text{ AaCoA}\left(t\right)$$

$$\frac{d\text{Mev}\left(t\right)}{dt}=\;-k_{HMGS}\text{ AaCoA}\left(t\right)-k_{MK}\text{Mev}\left(t\right)$$

$$\frac{d\text{Mev}5\text{P}\left(t\right)}{dt}=\;-k_{MK}\text{ Mev}\left(t\right)-\;k_{PMK}\text{Mev}5\text{P}\left(t\right)$$

$$\frac{d\text{Mev}5\text{PP}\left(t\right)}{dt}=\;-k_{PMK}\text{ Mev}5\text{P}\left(t\right)$$

Where *k*_*x*_ is the level of activity of the enzyme *x*, expressed as fmol/h/CA (S1 Table).

For the JH branch, data were available for the activities of 4 sequential enzymes, farnesyl diphosphate phosphatase (FPPase), farnesol oxidase (FOLSDR), farnesal dehydrogenase (FALDH) and juvenile hormone acid methyl transferase (JHAMT), which allowed us to model a segment that involves the conversion of farnesyl diphosphate to methyl farnesoate. The dependent variables in the model were FPP (farnesyl diphosphate), FOL (farnesol), FAL (farnesal), FA (farnesoic acid), and MF (methyl farnesoate). This set of ordinary differential equations (ODEs) can be written as:

$$\frac{d\text{FPP}\left(\text{t}\right)}{dt}=\;-k_{FPPase}\text{ FPP}\left(t\right)$$

$$\frac{d\text{FOL}\left(\text{t}\right)}{dt}=\;k_{FPPase}\text{FPP}\left(t\right)-k_{FOLSDR}\text{ FOL}\left(t\right)$$

$$\frac{d\text{FAL}\left(\text{t}\right)}{dt}=\;k_{FOLSDR}\text{ FOL}\left(t\right)-k_{FALDH}\text{ FAL}\left(t\right)$$

$$\frac{d\text{FA}\left(\text{t}\right)}{dt}=\;k_{FALDH}\text{ FAL}\left(t\right)-k_{JHAMT}\text{ FA}\left(t\right)$$

$$\frac{d\text{MF}\left(\text{t}\right)}{dt}=\;k_{JHAMT}\text{ FA}\left(t\right)$$

ODE systems were integrated numerically using the explicit method of Euler [24] with an integration time step of 10^-10^h implemented in Mathematica 9.1 (Wolfram Research).

## 3. Results

### 3.1. General additive models of the JH biosynthesis pathway

Changes in specific metabolite concentrations, as a result of changes in enzymatic activities and substrate concentrations, were estimated using GAMs based on experimental data from previous studies [10] (S1 Table). A summary of the statistics of the fitted models is presented for all the metabolites included in the MVAP (Table 1) and JH-branch (Table 2).

**Table 1:** Summary statistics of the fitted models applied for mevalonate pathway.

Model	Adj R^2^	Explained deviance (%)	AIC
**Acetoacetyl-CoA**			
*g*(*μ*_*i*_) = *α*	0	0	557.5
*g*(*μ*_*i*_) = *α* + *f*_1_(*Acetyl* − *CoA*)	0.04	7.0	522.9
**HMG-CoA**			
*g*(*μ*_*i*_) = *α*	0	0	319.2
*g*(*μ*_*i*_) = *α* + *f*_1_(*Acetoacetyl* − *CoA*)	0.18	21.7	315.1
*g*(*μ*_*i*_) = *α* + *f*_2_(*HMGS*)	0.25	34.9	314.6
*g*(*μ*_*i*_) = *α* + *f*_1_(*Acetoacetyl* − *CoA*) + *f*_2_(*HMGS*)	**0.55**	**62.6**	302.5
**Mevalonate**			
*g*(*μ*_*i*_) = *α*	0	0	630.3
*g*(*μ*_*i*_) = *α* + *f*_1_(*HMG* − *CoA*)	**0.43**	**52.4**	593.1
**Mevalonate-5P**			
*g*(*μ*_*i*_) = *α*	0	0	601.4
*g*(*μ*_*i*_) = *α* + *f*_1_(*Mevalonate*)	-0.02	0.8	603.2
*g*(*μ*_*i*_) = *α* + *f*_2_(*MK*)	0.15	**25.7**	599.2
*g*(*μ*_*i*_) = *α* + *f*_1_(*Mevalonate*) + *f*_2_(*MK*)	-0.02	4.0	604.1
**Mevalonate-5PP**			
*g*(*μ*_*i*_) = *α*	0	0	309.6
*g*(*μ*_*i*_) = *α* + *f*_1_(*Mevalonate* − 5*P*)	0.74	77.8	278.7
*g*(*μ*_*i*_) = *α* + *f*_2_(*PMK*)	0.21	32.1	306.2
*g*(*μ*_*i*_) = *α* + *f*_1_(*Mevalonate* − 5*P*) + *f*_2_(*PMK*)	**0.83**	**87.6**	269.1
**Isopentenyl-PP**			
*g*(*μ*_*i*_) = *α*	0	0	409.3
*g*(*μ*_*i*_) = *α* + *f*_1_(*Mevalonate* − 5*PP*)	-0.01	1.3	410.9
**Geranyl-PP**			
*g*(*μ*_*i*_) = *α*	0	0	687.5
*g*(*μ*_*i*_) = *α* + *f*_1_(*Isopentenyl* − *PP*)	0.10	**23.4**	616.9

**Table 2:** Summary statistics of the fitted models applied for JH branch.

Model	Adj R^2^	Explained deviance (%)	AIC
**Farnesyl-PP**			
*g*(*μ*_*i*_) = *α*	0	0	206.9
*g*(*μ*_*i*_) = *α* + *f*_1_(Geranyl − PP)	-0.05	0.0	208.9
*g*(*μ*_*i*_) = *α* + *f*_2_(*FPPS*)	0.68	74.4	186.9
*g*(*μ*_*i*_) = *α* + *f*_1_(Geranyl − PP) + *f*_2_(*FPPS*)	**0.84**	**88.2**	173.9
**Farnesol**			
*g*(*μ*_*i*_) = *α*	0	0	317.3
*g*(*μ*_*i*_) = *α* + *f*_1_(Farnesyl − PP)	0.14	18.6	313.9
*g*(*μ*_*i*_) = *α* + *f*_2_(*FPPase*)	0.04	7.8	317.0
*g*(*μ*_*i*_) = *α* + *f*_1_(Farnesyl − PP) + *f*_2_(*FPPase*)	**0.19**	**28.2**	313.7
**Farnesal**			
*g*(*μ*_*i*_) = *α*	0	0	500.3
*g*(*μ*_*i*_) = *α* + *f*_1_(Farnesol)	0.38	43.6	484.3
*g*(*μ*_*i*_) = *α* + *f*_2_(*FOL* − *SDR*)	0.05	8.06	499.0
*g*(*μ*_*i*_) = *α* + *f*_1_(Farnesol) + *f*_2_(*FOL* − *SDR*)	**0.37**	**44.6**	486.1
**Farnesoic acid**			
*g*(*μ*_*i*_) = *α*	0	0	509.9
*g*(*μ*_*i*_) = *α* + *f*_1_(Farnesal)	0.37	43.9	494.3
*g*(*μ*_*i*_) = *α* + *f*_2_(*FALDH*)	0.45	48.3	487.9
*g*(*μ*_*i*_) = *α* + *f*_1_(Farnesal) + *f*_2_(*FALDH*)	**0.45**	**49.6**	488.7
**Methyl farnesoate**			
*g*(*μ*_*i*_) = *α*	0	0	395.0
*g*(*μ*_*i*_) = *α* + *f*_1_(Farnesoic acid)	0.35	43.7	384.9
*g*(*μ*_*i*_) = *α* + *f*_2_(*JHAMT*)	0.19	27.8	390.8
*g*(*μ*_*i*_) = *α* + *f*_1_(Farnesoic acid) + *f*_2_(*JHAMT*)	**0.74**	**81.1**	359.0
**Juvenile Hormone III**			
*g*(*μ*_*i*_) = *α*	0	0	503.2
*g*(*μ*_*i*_) = *α* + *f*_1_(Methyl farnesoate)	**0.78**	**81.3**	427.2

The amounts of products generated by the seven enzymatic reactions included in the MVAP were modeled as a function of: 1) substrate, 2) enzymatic activity and, 3) the combination of substrate and enzymatic activity (Table 1). The adjusted R^2^, explained deviance (%) and Akaike information criterion (AIC) scores were calculated for each model. Due to the lack of enzymatic activity data, four of the models (acetoacetyl-CoA, mevalonate, isopentenyl-PP and geranyl-PP) were constructed using only substrate concentrations. From those four models, the synthesis of mevalonate showed the highest adjusted R^2^ (0.43) and best fit explained deviance (52.4%).

For the MVAP models in which data for both variables were available, the highest adjusted R^2^ was observed when both substrate and enzymatic activity were considered in the model; with explained deviances above 60% for HMG-CoA and Mev5PP. Furthermore, both models showed the lowest AIC values, suggesting that the models constructed were the most parsimonious for the given data. In contrast, the models built for Mev5P showed the lowest adjusted R^2^ and explained deviance, as well as the highest AIC, indicating that these models did not have significant statistical support for the given data, and pointing that synthesis of Mev5P may be strongly influenced by other factors.

For the JH-branch, six enzymatic reactions were modeled (Table 2). Models from the first five metabolites (FPP, FOL, FAL, FA and MF) were constructed using data on substrate concentration and enzymatic activities. The transformation of MF into JH was only modeled as a function of substrate due to the lack of experimental data on epoxidase activity. The model for the production of FPP by FPPS showed the highest adjusted R^2^ value, as well as the major explained deviance (88%); followed by the models constructed for JH (81.3%), MF (81.1%), FA (49.5%) and FAL (44.6%). The only bad-fitted model from the JH-branch was the FOL model, with an adjusted R^2^ of 0.19 and deviance explaining 28.2% of the data.

### 3.2. Interpretations of the GAMs

**Fig 2:**
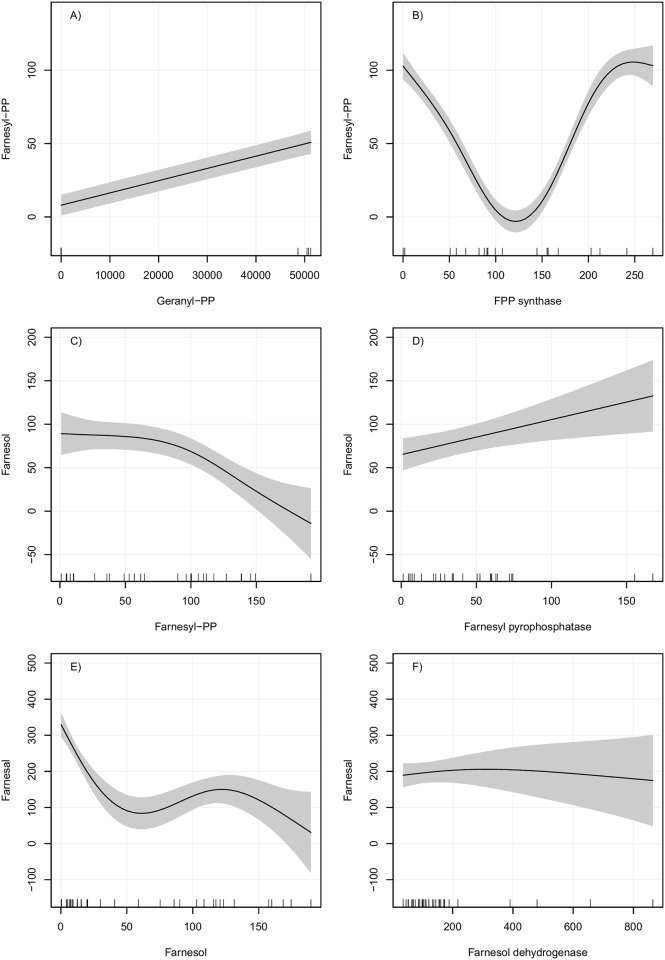
Partial dependence plots of GAMs for FPP, FOL and FAL production. Left panel: X axis is the substrate and Y axis the metabolite synthesized (product of reaction). Right panel: X axis is the enzymatic activity and Y axis the metabolite synthesized. All data are on fmol concentrations. Gray area indicates a 95% confidence interval for the smoothed lines. Lines on the bottom of the X axis indicate the observed values of the predictor variable.

**Fig 3:**
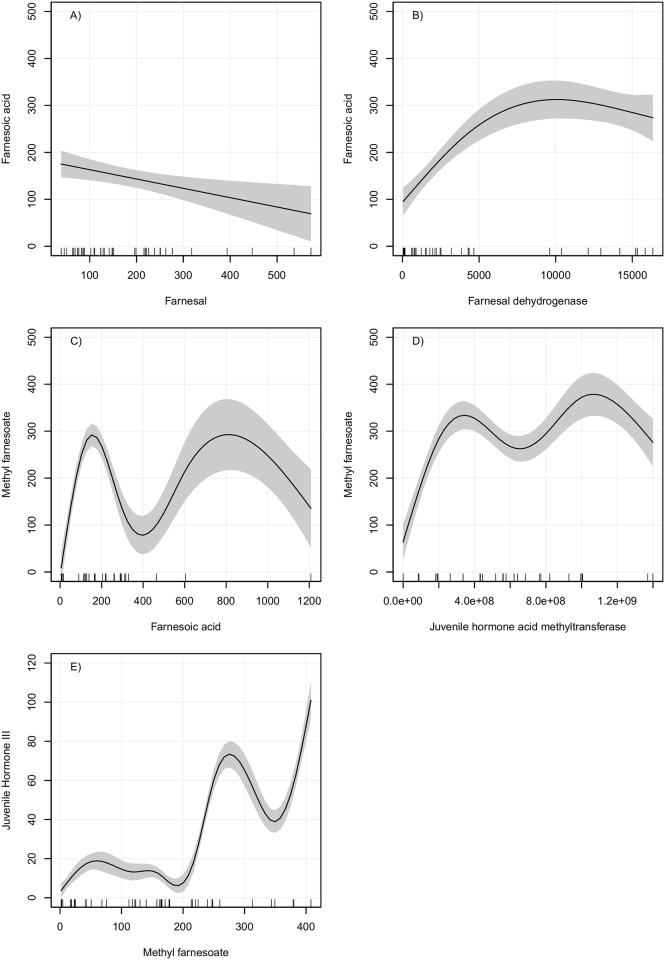
Partial dependence plots of GAMs for FA, MF and JH III production. Left panel: X axis is the substrate and Y axis the metabolite synthesized (product of reaction). Right panel: X axis is the enzymatic activity and Y axis the metabolite synthesized. All data are on fmol concentrations. Gray area indicates a 95% confidence interval for the smoothed lines. Lines on the bottom of the X axis indicate the observed values of the predictor variable.

Partial dependence plots were constructed to describe the relationships between substrates and reaction products, as well as enzyme activities and reaction products for the MVAP (S1 Fig) and JH-branch (Figs 2 and 3). The best-fitted models for the MVAP metabolites showed two different patterns: 1) an oscillatory dynamic behavior (e.g. mevalonate), and 2) a hyperbolic pattern (e.g. Mev5PP). The oscillatory pattern observed on the mevalonate model suggests that there is a substantial basal level of mevalonate production, which eventually rises as a consequence of increased concentrations of substrate (S1 Fig). The relative low score of the model (substrate vs. product) suggests than changes in enzymatic activities play more important roles than substrate concentration on the mevalonate synthesis. On the contrary, the production of Mev5PP is mainly governed by increases in substrate concentration until reaching a high threshold (S1 Fig), since its model is well supported by changes in substrate concentration (0.74), with the addition of the enzymatic activity data contributing only with an increase of 0.09 in the fitness of the model (0.83).

The best-fitted models for JH-branch metabolites are shown in Figs 2 and 3. Two different patterns were observed for models in which only substrate and product concentrations were considered: 1) an inverse relationship and 2) an oscillatory dynamic behavior. The production of FAL presented an oscillatory model when substrate concentration was considered (Fig 2E). When the enzymatic activity of FOLSDR was used, the partial dependence plot for FAL indicated that the reaction was not primarily affected by changes in enzymatic activities, but other factors that were not plotted were certainly influencing the synthesis of FAL.

The FA model showed that higher concentrations of FAL did not lead to the production of more FA, which might be related to the toxicity of FAL in cells (Fig 3A). On the other hand, it is clear, that higher activities of farnesal dehydrogenase (FALDH) resulted in major productions of FA (Fig 3B).

The MF model displayed two potential thresholds that might affect its synthesis; one based on substrate concentration (Fig 3C), and a second on JHAMT activity (Fig 3D). In both cases, the models predicted oscillatory increases in the production of MF. Interestingly, the JH III model in which only the substrate concentration was considered, displayed similar high fitness score than the MF model, suggesting that there is significant correlation between MF and JH III concentrations. The JH III model implies that elevated production of JH III (over 80 fmol) were only achieved with high concentrations of MF (over 350 fmol).

### 3.3. GAM validations

**Fig 4:**
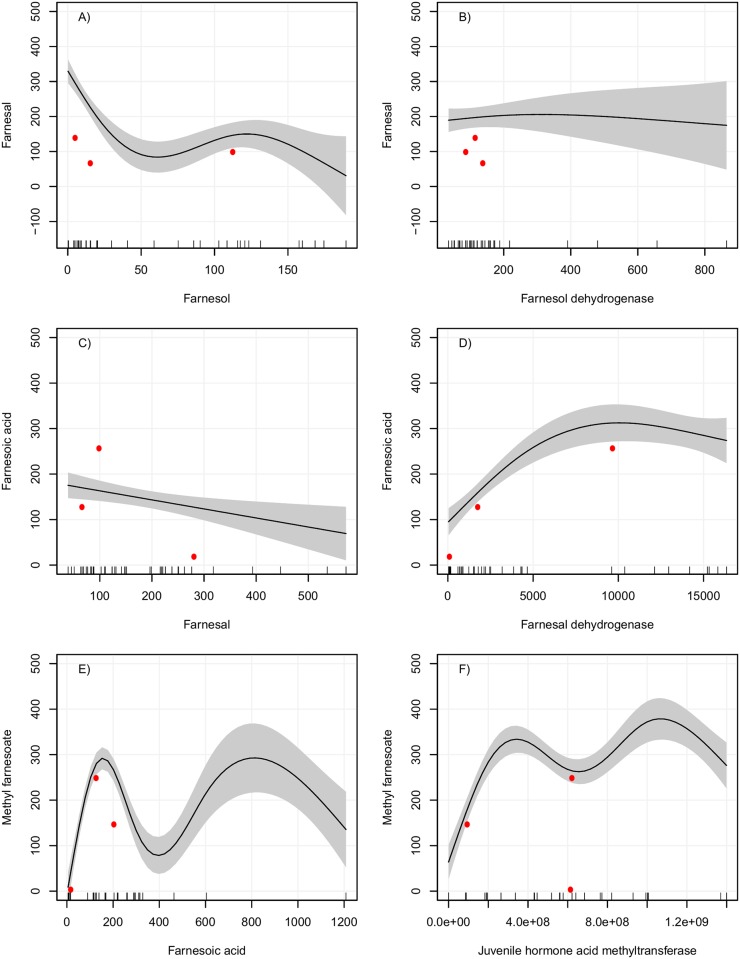
Partial dependence plots for the validation of GAMs for FAL, FA and MF production. Left panel: X axis is the substrate and Y axis the metabolite synthesized (product of reaction). Right panel: X axis is the enzymatic activity and Y axis the metabolite synthesized. All data are on fmol concentrations. Gray area indicates a 95% confidence interval for the smoothed lines. Lines on the bottom of the X axis indicate the observed values of the predictor variable. Red dots indicate data not used for the model construction.

Validation of the models were performed using experimental data that were not included in the building of the model (S2 Table). Predicted values for the FA, FAL and MF models are shown in Fig 4. Most of the predicted values were within the estimated confidence interval shown in the plots, suggesting that the models can be used to predict the behavior of the intermediates in the JH-branch.

### 3.4. Ordinary Differential Equations models

**Fig 5:**
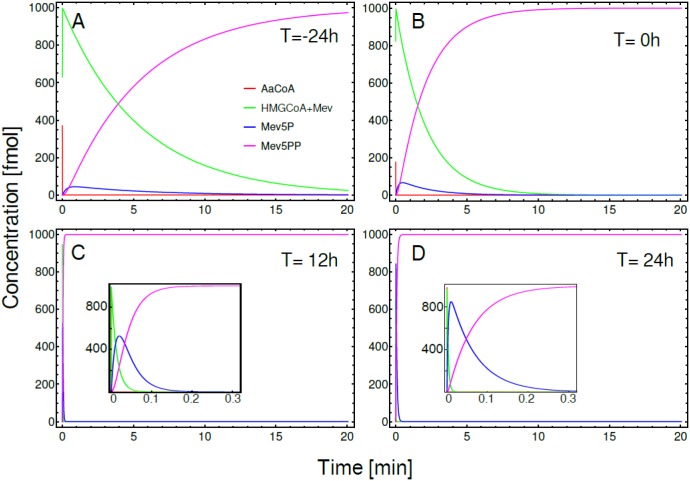
ODE simulations of a defined segment of the MVAP. Temporal evolution of the concentrations of AaCoA (red line), HMGCoA + Mev (green), Mev5P (blue line) and Mev5PP (magenta line). Simulation of CA activities in: A) Female pupae, 24h before adult eclosion. B) Female at adult eclosion (0h). Sugar-fed female at C) 12h and D) 24h after adult eclosion. Inset in panels C and D: details of pathway dynamics at higher temporal resolution. In all these simulations the initial concentration of precursor (AaCoA) was 1000 fmol, while all other initial concentrations were zero. Simulations were run for 20 min.

The temporal changes in the concentration of precursors, intermediate metabolites and final products, in a defined segment of the MVAP, were obtained by the numerical integration of ODEs; using enzyme activities from four biologically relevant different conditions: female pupae 24h before adult eclosion (Fig 5A), newly eclosed adult female (Fig 5B), and sugar-fed females 12h and 24h after adult eclosion (Fig 5C and 5D, respectively). We used the same amount of the original precursor (AaCoA = 1000 fmol) as the initial condition in the four models, which were run for 20 min. Our simulations indicated that 24h before eclosion the rate of change in the concentrations were slow, in agreement with the low activity of JH synthesis by the pupae CA (Fig 5A). The first step in the segment, the conversion of AaCoA into HMG-CoA, was very fast when compared with the subsequent reactions. On the other hand, the conversion into Mev5P was quicker than its transformation into the final product (Mev5PP). This last conversion was the rate-limiting step in this section of the MVA pathway, with most of the initial precursor transformed into Mev5PP in around 20 minutes. At the end of the pupal stage (0h) the conversion into Mev5P and its subsequent transformation into Mev5PP have increased their kinetic rates around two-folds (Fig 5B). After adult eclosion there is a generalized increment in the activity level of all the enzymes, and the time needed to reach the final concentration of Mev5PP, was only 10 seconds (Fig 5C and 5D). We also detected an increment in the activity of the mevalonate kinase in relation to the activity of the phosphomevalonate kinase, which resulted in the transient accumulation of Mev5P. This accumulation was more evident 24h after adult eclosion (inset of Fig 5D).

**Fig 6:**
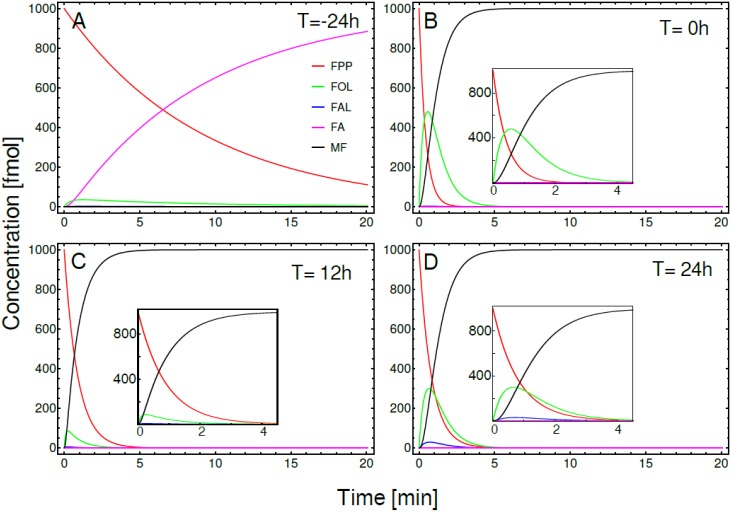
ODE simulations of a defined segment of the JH-branch pathway. Temporal evolution of the concentrations of FPP (red line), FOL (green line), FAL (blue line), FA (magenta line) and MF (black line). Simulation of CA activities in: A) Female pupae, 24h before adult eclosion. B) Female at adult eclosion (0h). Sugar-fed female at C) 12h and D) 24h after adult eclosion. Inset in panels B, C and D: details of pathway dynamics at higher temporal resolution. In all these simulations the initial concentration of precursor (FPP) was 1000 fmol, while all other initial concentrations were zero. Simulations were run for 20 min.

We also used ODEs to model a segment of the JH branch. The time course of the changes in the concentration of the initial precursor (FPP, red line), intermediate metabolites and the final product (MF, black line) are depicted in Fig 6. These concentrations were obtained by numerical integration of the ODE system in the same four biological conditions described for the MVAP simulations: 24h before adult eclosion (Fig 6A), newly eclosed adult (Fig 6B), and sugar-fed females 12h and 24h after adult eclosion (Fig 6C and 6D, respectively). For these models, we used 1000 fmol of FPP as the initial precursor. In the pupae 24h before eclosion all the enzymatic activities were very low, and the time needed to reach the steady state concentration of FA was over the 20 minutes that lasted the simulation. The enzymatic activity of JHAMT was almost undetectable; consequently, the concentration of MF was also too low to detect (Fig 6A). This landscape changed dramatically after eclosion, when there is a remarkable increase in the activity of JHAMT, as well of the activities of all the other JH-branch enzymes. As a consequence of these higher enzymatic activity levels, the steady state concentration of the final product (MF) were reached in around 5 min. After adult eclosion the conversion of FA into MF was very fast when compared with the other metabolic steps, which lead to a fast depletion of the FA pool (Fig 6B, 6C and 6D). Twenty-four hours after eclosion, a reduction in the activity of farnesal dehydrogenase made possible to observe a transient small accumulation of FAL, which was rapidly metabolized to FA (Fig 6D).

**Fig 7:**
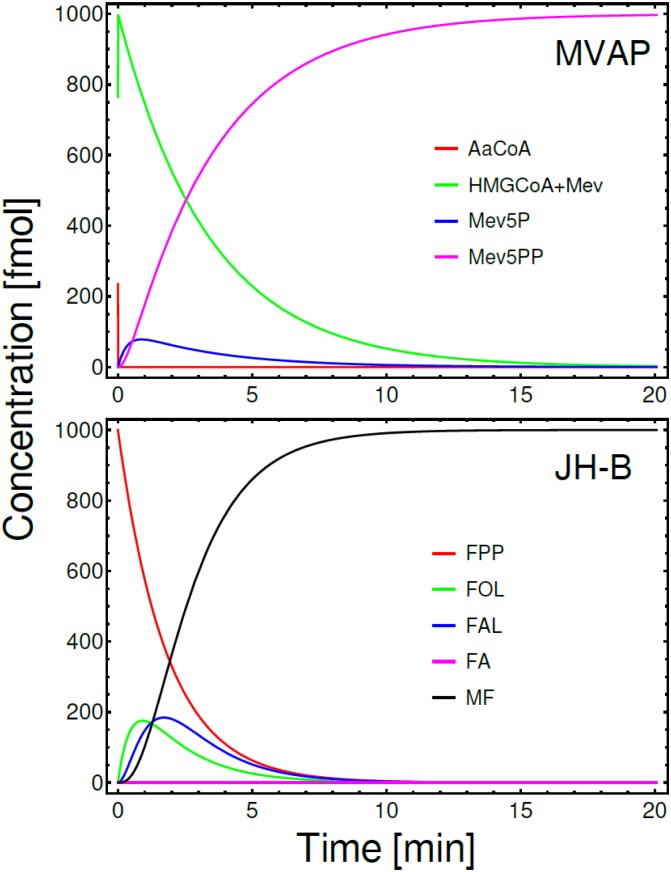
ODE simulations of defined segments of the MVAP and JH-branch 24h after blood feeding. Temporal evolution of the concentrations of metabolites are as Figs 5 and 6. In all these simulations the initial concentration of precursor (AaCoA or FPP) were 1000 fmol, while all other initial concentrations were zero. Simulations were run for 20 min.

We also simulated the changes in these same segments of the MVAP and JH-branch using the enzymatic activity levels recorded 24h after blood feeding, when the CA activity is greatly depressed. Fig 7 shows the results of these simulations, where one can see an evident reduction of the overall output of the two pathways.

## 4. Discussion

### 4.1. Juvenile hormone pathway architecture

It is challenging to construct accurate models for metabolic pathways that can be rigorously fitted to experimental data. Pathway modelling has been extensively used to understand prokaryotic metabolism [25], but remains largely undeveloped for eukaryotic systems.

The importance of JH in the regulation of development and reproduction in mosquitoes stimulated the study of the biosynthetic pathway, and resulted in the collection of unique experimental data on changes in the levels of metabolites, as well as information on enzymatic activities from CA extracts [10]. These types of data were used in this study to model the enzymatic reactions included into the JH biosynthetic pathway in the CA of female *A*. *aegypti* mosquitoes.

The dynamic JH III pathway encompasses changes in 14 metabolites catalyzed by the activities of 13 enzymes, that are linked in a linear track (Fig 1). Acetyl-CoA molecules feed the pathway, and after transforming into 12 different compounds, exit the CA as JH III. In our models, we tried to predict the changes in the formation of each of these 13 compounds, as the result of changes in the amounts of their correspondent substrates, as well as the fluctuations in specific catalytic enzymatic activities. Each reaction, although modeled individually, is integrated into the pathway, and therefore influenced by other factors, besides the specific changes in substrate concentration and the activity of a particular enzyme. A few premises need to be accepted when building the GAMs and ODEs, 1) the pathway is linear, 2) all substrates are available to their respective enzymes, and 3) cofactor concentrations are not limiting the reactions.

An important point at issue is the linearity of the pathway; the MVAP consists of a main trunk followed by sub-branches that produce a diverse range of compounds; branch point regulation is an important mechanism controlling carbon flow in the MVAP; the FPP produced by the MVAP can be shunted to many metabolic branches for the synthesis of critical molecules such as ubiquinone, dolichol, or prenylated proteins [3]. Insects lack the cholesterol-synthetic branch present in vertebrates, but in the CA the MVAP branches into the synthesis of JH. Because the CA is a specialized site of production of JH, branching of MVAP into non JH molecules is probably very minor quantitatively, at least during periods of high JH synthesis. An additional branch is the IPP- DMAPP branch, as the sesquiterpenoid is built from one starting block and two additions. So one third of the IPP is diverted to DMAPP, which could lead to complications of numerical models.

In addition to branching, we have documented in the CA of mosquitoes the existence of at least one reversal of flux directionality (farnesal back to farnesol) [5], as well as feed-back loops [26]; therefore, the absolute linearity of the pathway is questionable.

### 4.2. Precursor pools changes

In the JH biosynthetic pathway, the 13 enzymes are connected by metabolite pools that are common to them, for example FOL is the product of FPPase activity and the substrate for farnesol dehydrogenase. The pools are the links in the system interactions, consequently pool concentrations and fluxes (which are flows into and out of pools) are vital variables in JH regulation. Just about adult eclosion, when JH synthesis increases, the fast metabolism of MVAP precursors results in a depletion of MVAP intermediate pools [10] (S1 Table). These declines in precursors were reflected into partial dependence plots where maximum production of metabolites, such as Acetoacetyl-CoA or HMG-CoA were observed in the misleading “absence” of their respective substrates (negative or inverted relationships). In reality, what we were detecting was the fast catalysis and lack of accumulation of those two metabolites (Acetyl-CoA and Acetoacetyl-CoA respectively). As changes in pathway are fast, a shorter temporal sampling of the enzymes activities could change the reported negative relationships. It is important to emphasize that the experimental values for the pool sizes are revealing only the amount of metabolites that we were able to detect in CA extracts; they are not describing the amount of precursor available to the corresponding enzyme.

There are other factors that might add additional levels of complexity that affect model interpretations, such as the compartmentalization of metabolite pools; we have described that the CA of a newly emerged female mosquito, which has a very large FA pool but limited JH synthesis, can be strongly stimulated by exogenous supply of FA [9, 10]. These results suggest differences in the channeling of “endogenous” and “exogenous” FA derived pools, and possibly other precursor pools. MF is another example of pool compartmentalization, it has extremely low solubility, so it probably partitions into the ER membrane, where it is metabolized by the epoxidase. Consequently, there is likely not a direct relation between MF levels in the CA and MF concentration as substrate for the epoxidase. These has been experimentally validated for the MF epoxidase in the locust *Locusta migratoria* [27].

In addition, there is at least one example of a reversal of the flux in the JH synthesis pathway in mosquitoes that also affects pool sizes; specifically, the deficiency of farnesal dehydrogenase activity at 24 h after blood feeding results in a large increase of the FAL pool that cannot be converted into FA. Consequently, the accumulation of the potentially toxic FAL stimulates the activity of a reductase that converts FAL back into FOL, resulting in FOL leaking out of the CA [5]. Again, these metabolic features are evident into partial dependence plots, where maximum production of FAL was observed in the “absence” of FOL (negative or inverted relationships); while in fact what we were detecting was the normal generation of FOL, accumulation of FAL, followed by a reversal of the flux with disposal of the newly generated FOL.

Furthermore, in the CA of mosquitoes, some MVAP precursor pools are controlled by feedback regulation imposed by metabolites upwards in the pathway; such as the example of GPP and FPP having a negative feedback on the activity of mevalonate kinase, and therefore affecting the mevalonate and mevalonate-5P pools [26]. In summary, despite these limitations, our GAMs confirmed that availability of endogenous intermediates is one of the most critical parameter for the regulation of the total flux in the pathway, with changes in substrate concentrations explaining 50% or more of the model deviances in 7 of the 13 metabolic steps analyzed.

### 4.3. Enzymatic activities changes

The activities of 8 of the 13 JH biosynthetic enzymes have been monitored *in vitro* in CA extracts of mosquitoes under 5 different developmental and physiological conditions [10]. The catalytic activities of the enzymes of the MVAP and JH-branch changed in a coordinated fashion in the “active” and “inactive” CA. Changes in enzymatic activities were generally concurrent with increases or decreases in JH synthesis, but it is less clear how much these changes in enzymatic activities are affecting the flux in the pathway.

In physiological systems, enzymatic flux capacities (enzyme *V*_*max*_ values) are often in excess of maximum physiological flux rates or loads (*v*) [28]. It has been proposed that in a synthetic pathway containing numerous enzymes, almost all the enzymes will appear to be “in excess”, in the sense that individual quantities or activities can be considerably reduced without appreciable effect on the flux [29]. This seems to be the case for the enzymes of the JH synthesis pathway; flux control theory was used to correlate the effect of mevinolin, a potent competitive inhibitor of HMG-CoA reductase on reductase activity and JH III synthesis in the CA of *Diploptera punctata* cockroaches [30]; results indicated that HMG-CoA reductase has a low control coefficient on JH III synthesis both on CA with high and low rates of JH synthesis. HMG-CoA synthase [31] and HMG-CoA reductase [32] activities change over 6- to 10-fold over the gonotrophic cycle of *D*. *punctata*. The mean activities of these two enzymes during the peak of JH synthesis were in large excess of the rate needed to account for the level of JH synthesis by CA of that age [31, 32]. Thus, the patterns of HMG-CoA synthase and HMG-CoA reductase changes during the cycle of activity, and the effects of inhibitors all argue against a rate-limiting role of any of these two enzymes in JH III biosynthesis. Stimulation of JH synthesis with exogenous precursors has been reported for the CA of many insect species, and it seems that having an excess of enzymes is common in most insects studied. In *A*. *aegypti*, the individual addition of 200 μM of any of 9 different precursors (ACoA, MVA, MevP, MevPP, FPP, FOL, FAL, FA and MF) resulted in a stimulation of 2–3 fold on JH synthesis [33]; confirming that enzymatic flux capacities were higher that the basal flux rates observed in controls.

It is important to point out that we have observed striking differences in the levels of activities among the 8 enzymes studied; with HMGS and JHAMT displaying activities in the high nanomolar range (500–1000 nmol/h), while FPPase maximum values were merely around 75 fmol/h. In addition, the activity of a particular enzyme, such as JHAMT, fluctuated between 200 fmol/h and 1400 nmol/h. Nevertheless, enzymatic activity levels need to surpass minimum thresholds to achieve a net flux of precursors through a biosynthetic pathway [29, 34]. Although our GAMs confirmed that availability of endogenous intermediates is the most critical parameter for the regulation of the total flux in the pathway, the statistical values of the models (R^2^, deviances and AIC scores) showed in some cases better fitness for models based on changes in enzymatic activities (HMG-CoA, Mev-5P, FPP and FA), than models based on substrate concentration. On the other hand, addition of information on enzymatic activities usually improved the fitness of models built solely based on substrate concentrations.

### 4.4. What can we learn from these models?

In biochemical models, when K_m_ is similar to S, then *v* will be directly related to S; but when S is much larger, *v* is limited by V_max_. Our models are not explicitly biochemical models, where we could determine and calculate K_m_ and V_max_ by relating enzymatic reaction rates (*v*) to substrate concentrations [S]. In contrast, they are kinetic models, where V_max_ is an approximation to enzyme concentration [E], and pools sizes of substrates are considered concentrations of substrate [S].

Our models evaluated how changes in substrate and enzymatic activities affected product yielding. High scores from the GAM models indicated that there were reactions from the MVAP and JH-branch that were mainly governed by changes in the availability of substrate and/or enzymatic activity. On the contrary, reactions showing less than a 50% explained deviance, suggested that additional factors might play more significant roles controlling these reactions.

Several cofactors are critical for JH synthesis (Fig 1), such as ATP, NAD^+^, NADP^+^, NADPH^+^, Mg^2+^ and S-adenosine methionine (SAM). Nicotinamide adenine dinucleotide coenzymes are essential for several enzymatic steps in the pathway, and their pool sizes can also be limiting factors in the mosquito CA [7, 5]; however, Diploptera CA shunted one third of the glucose provided through the pentose pathway (that generate NADPH), suggesting that unlikely this cofactor would be a limiting factor in the cockroach [35]. Reductions in the size of the ATP pool could also have detrimental effects on JH synthesis, since 9 molecules of ATP are used to build each FPP molecule. The FPPase activity in the mosquito CA is Mg^2+^ dependent; and FPPS also displays its activity only in the presence of metal cofactors; with the product condensation depending on the divalent cation. Mg^2+^ ions lead to the production of FPP, while the presence of Co^2+^ ions lead to GPP production [36].

SAM is a critical cofactor for JHAMT activity [4]. CA incubated in methionine-free medium synthesize only a small amount of JH III; showing that the intra-glandular pool of methionine is minute. There is an absolute dependence of JH III synthesis on exogenous methionine supply, with increases in the concentration of methionine in the incubation medium resulting in a linear increase in JH III synthesis [37]. We did not have access to experimental values on changes in the availability of all these cofactors, therefore these data were not included in the models; adding any of them as new predictor variables most likely would increase the fitness of the GAMs.

Several GAMs showed oscillatory dynamic behaviors. Enzymatic systems may display rhythmic behavior in two types of circumstances, which may be referred to as “endogenous” or “exogenous”, depending on whether the rhythm originates from within the system or through its coupling with some external periodic process. In other words, either changes in enzymes and substrate concentrations are at the core of the oscillatory mechanism, or they are driven by some external oscillation [38]. Oscillations might occur as a result of feedback regulation, driven by cofactor fluctuations or changes in the activity of regulatory molecules such as allatostatin-C [33]. Those factors affect the amplitude, period, and waveform of the oscillations produced by changes in substrate or enzymatic activity. These oscillations occur in a range bounded by two critical threshold values, in some cases (MF and JH III production), we observed explosive synthesis of the product occurring as soon as the substrate exceeds a threshold. Enzyme saturation eventually puts an upper bound on the enzyme reaction rate. Oscillations in enzymatic activities could be controlled through phosphorylation–dephosphorylation, through enzyme synthesis and degradation, and through association with protein inhibitors [38].

### 4.5. Conclusions

In previous studies, principal component analysis (PCA) of the metabolic pools indicated that in reproductive female mosquitoes, at least four developmental switches alter JH synthesis by modulating the flux of isoprenoids at distinct points. Metabolic analysis delimited four distinct CA physiological conditions that were named: inactive, active, modulated and suppressed CA [10]. The molecular bases for regulation of JH synthesis, as well as the roles of brain factors or other endocrine regulators changed during these four phases. Modeling the JH pathway can help to integrate experimental knowledge into a coherent picture and to test and/or support hypothesis about different mechanisms of JH synthesis regulation. In these models, we were able to integrate changes in precursors and enzymes involved in the synthesis of each intermediate metabolite for the JH synthesis. We tested the model by using experimental data and proved that the best fitted models can be used to predict the level of metabolites for *in vitro* experiments. Although limited by the absence of a complete set of experimental data, our ODEs underscored that in a sugar-fed female enzymatic activities were not limiting. This was also suggested by the GAMs that showed that changes in substrate concentrations explained 50% or more of the model deviances in 7 of the 13 metabolic steps analyzed, in other words substrate concentrations are primarily limiting the flux. In addition, when comparing the sizes of the intermediate pools (100–1000 fmol/CA) with the activities of most of the enzymes (103–106 fmol/CA/h), the enzymatic activities are in excess and therefore the processing of the substrates is often very fast.

Prediction of the output of metabolic pathways by modelling their reaction networks is important because pathways provide a higher level of organization that facilitate the comprehension of the network, allowing the calculation *in silico* of the effect of potential disturbances on the enzymes or metabolites in the JH pathway.

## Supporting information

S1 Fig Partial dependence plots for GAMs for MVAP metabolites production. Left panel: X axis is the substrate and Y axis the metabolite synthesized (product of reaction). Right panel: X axis is the enzymatic activity and Y axis the metabolite synthesized. All data are on fmol concentrations. Gray area indicates a 95% confidence interval for the smoothed lines. Lines on the bottom of the X axis indicate the observed values of the predictor variable.
(TIF)

S1 Table Experimental data on enzymatic activities and CA metabolite concentrations, used for model building. Data are from female pupae and adult *Aedes aegypti* mosquitoes, and were obtained as previously reported [Rivera-Perez C, Nouzova M, Lamboglia I, Noriega FG. Metabolic analysis reveals changes in the mevalonate and juvenile hormone synthesis pathways linked to the mosquito reproductive physiology. Insect Biochem. Molec. Biol. 2014; 51:1–9.]. Enzymatic activities were measured using “*in vitro*” assays that evaluated the capacity of CA extracts to metabolize MVAP and JH-branch intermediates, and are expressed as fmol/h/CA. Metabolite concentrations are the sizes of the precursor pools detected in CA extracts, and expressed as fmol/CA.
(XLSX)

S2 Table Experimental data on enzymatic activities and CA metabolite concentrations, used for validation analyses. Data are from female pupae and adult *Aedes aegypti* mosquitoes, and were obtained as previously reported [Rivera-Perez C, Nouzova M, Lamboglia I, Noriega FG. Metabolic analysis reveals changes in the mevalonate and juvenile hormone synthesis pathways linked to the mosquito reproductive physiology. Insect Biochem. Molec. Biol. 2014; 51:1–9.]. Enzymatic activities were measured using “*in vitro*” assays that evaluated the capacity of CA extracts to metabolize MVAP and JH-branch intermediates, and are expressed as fmol/h/CA. Metabolite concentrations are the sizes of the precursor pools detected in CA extracts, and expressed as fmol/CA.
(XLSX)
