# Supplementary figures and images for: Modeling the flux of metabolites in the juvenile hormone biosynthesis pathway using generalized additive models and ordinary differential equations
Source: PLoS One. 2017 Feb 3;12(2):e0171516. doi: 10.1371/journal.pone.0171516 (PMC5291429; doi:10.1371/journal.pone.0171516)

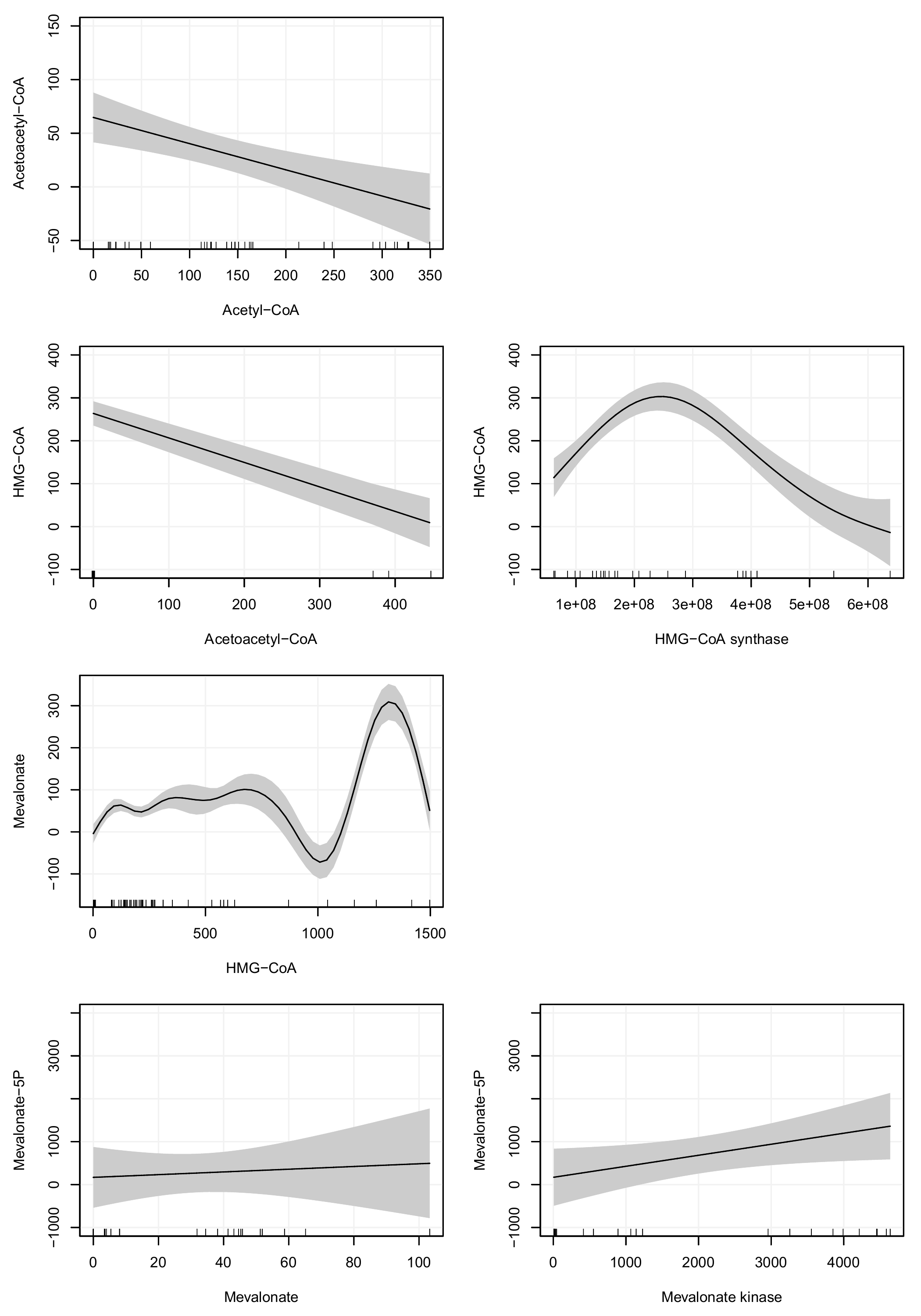

Supplement: S1 Fig — Left panel: X axis is the substrate and Y axis the metabolite synthesized (product of reaction). Right panel: X axis is the enzymatic activity and Y axis the metabolite synthesized. All data are on fmol concentrations. Gray area indicates a 95% confidence interval for the smoothed lines. Lines on the bottom of the X axis indicate the observed values of the predictor variable. (TIF) [file pone.0171516.s001.tif]
